# Changing epidemiology of *Plasmodium vivax* malaria in Nouakchott, Mauritania: a six-year (2015–2020) prospective study

**DOI:** 10.1186/s12936-023-04451-3

**Published:** 2023-01-17

**Authors:** Inejih El Moustapha, Jemila Deida, Mariem Dadina, Abdellahi El Ghassem, Mariem Begnoug, Mariem Hamdinou, Khadijetou Mint Lekweiry, Mohamed Salem Ould Ahmedou Salem, Yacoub Khalef, Amal Semane, Khyarhoum Ould Brahim, Sébastien Briolant, Hervé Bogreau, Leonardo Basco, Ali Ould Mohamed Salem Boukhary

**Affiliations:** 1 Unité de Recherche Génomes et Milieux (GEMI), Université de Nouakchott, Nouveau Campus Universitaire, BP 5026, Nouakchott, Mauritania; 2 Unité de Recherche Ressources Génétique et Environnement, Institut Supérieur d’Enseignement Technologique (ISET), Rosso, Mauritania; 3 Department of Pediatrics, Mother and Children Hospital Centre, Centre Hospitalier Mère et Enfant (CHME), Nouakchott, Mauritania; 4 Teyarett Health Centre (Centre de Santé de Teyarett), Nouakchott, Mauritania; 5 Aix Marseille Univ, IRD, AP-HM, SSA, VITROME, Marseille, France; 6 IHU-Méditerranée Infection, Marseille, France; 7 Unité de Parasitologie Entomologie, Département de Microbiologie et Maladies Infectieuses, Institut de Recherche Biomédicale des Armées (IRBA), Marseille, France

**Keywords:** *Anopheles arabiensis*, Climate changes, Diagnosis, Duffy antigen, Epidemiology, Malaria, *Plasmodium falciparum*, *Plasmodium vivax*, Polymerase chain reaction, Primaquine, Water distribution

## Abstract

**Background:**

*Plasmodium vivax* malaria is one of the major infectious diseases of public health concern in Nouakchott, the capital city of Mauritania and the biggest urban setting in the Sahara. The assessment of the current trends in malaria epidemiology is primordial in understanding the dynamics of its transmission and developing an effective control strategy.

**Methods:**

A 6 year (2015–2020) prospective study was carried out in Nouakchott. Febrile outpatients with a clinical suspicion of malaria presenting spontaneously at Teyarett Health Centre or the paediatric department of Mother and Children Hospital Centre were screened for malaria using a rapid diagnostic test, microscopic examination of Giemsa-stained blood films, and nested polymerase chain reaction. Data were analysed using Microsoft Excel and GraphPad Prism and InStat software.

**Results:**

Of 1760 febrile patients included in this study, 274 (15.5%) were malaria-positive by rapid diagnostic test, 256 (14.5%) were malaria-positive by microscopy, and 291 (16.5%) were malaria-positive by PCR. *Plasmodium vivax* accounted for 216 of 291 (74.2%) PCR-positive patients; 47 (16.1%) and 28 (9.6%) had *P. falciparum* monoinfection or *P. vivax*–*P. falciparum* mixed infection, respectively. During the study period, the annual prevalence of malaria declined from 29.2% in 2015 to 13.2% in 2019 and 2.1% in 2020 (*P* < 0.05). Malaria transmission was essentially seasonal, with a peak occurring soon after the rainy season (October–November), and *P. vivax* infections, but not *P. falciparum* infections, occurred at low levels during the rest of the year. The most affected subset of patient population was adult male white and black Moors. The decline in malaria prevalence was correlated with decreasing annual rainfall (r = 0.85; *P* = 0.03) and was also associated with better management of the potable water supply system. A large majority of included patients did not possess or did not use bed nets.

**Conclusions:**

Control interventions based on prevention, diagnosis, and treatment should be reinforced in Nouakchott, and *P. vivax*-specific control measures, including chloroquine and 8-aminoquinolines (primaquine, tafenoquine) for treatment, should be considered to further improve the efficacy of interventions and aim for malaria elimination.

## Background

Despite considerable multisectoral, international efforts to control and eliminate malaria since the end of the Second World War, malaria is still one of the most devastating infectious diseases worldwide, particularly in the tropics. According to the latest World Malaria Report 2022, malaria is responsible for an estimated 247 million cases globally, of which 95% are reported in Africa [1]. The total number of malaria deaths in 2021 was estimated to be 619,000, with 96% occurring in Africa [1].

Most malaria cases in endemic areas around the world are caused by *P. falciparum* or *P. vivax* [2, 3]. Both of these species are present in Asia, Central and South America, western Pacific region, and East Africa (mostly in the Horn of Africa and Madagascar), with varying proportions between these species, depending on the endemic areas and intensity of malaria control interventions. In Africa, where *P. falciparum* and *P. vivax* co-exist, *P. falciparum* is generally more prevalent than *P. vivax* [4, 5]. A large majority of malaria-associated deaths are due to *P. falciparum* [2]. *Plasmodium vivax* can also lead to severe complications resulting in death but much less frequently than *P. falciparum* [6].

Malaria continues to be one of the major health issues in Mauritania [7]. Malaria transmission is seasonal and intense in the southern Sahelian zone and seasonal and unstable in the northern Saharan zone [8–11]. *Plasmodium falciparum* and *P. vivax* co-exist in Mauritania, but *P. falciparum* predominates in the Sahelian zone and *P. vivax* prevails in the Saharan zone [9–15]. Nouakchott, the capital city of Mauritania, is situated in the Saharan zone, where the majority of laboratory-confirmed malaria cases are due to *P. vivax* [12, 16]. Because of the short rainy season (July–September), the peak of malaria transmission often occurs during the months of October and November, immediately following the rainy season [8]. Some *P. vivax* cases have also been diagnosed during the dry season, most probably due to relapses arising from persistent dormant liver stages (i.e., hypnozoites) of the parasite [13]. Few cases of laboratory-confirmed *P. falciparum* have also been reported during the transmission period, mostly in individuals with a recent travel history to the southern and southeastern regions of the country where *P. falciparum* is highly endemic [8]. *Anopheles arabiensis* is the primary malaria vector in Nouakchott [17]. The presence of man-made water bodies, such as pools around public standpipes and household water storage tanks, has been conducive to the creation of *An. arabiensis* larval habitats in the capital city [18].

The last published epidemiological data on malaria cases, as well as assessment of therapeutic efficacy of chloroquine against *P. vivax*, in Nouakchott date back to 2013, the year when *P. vivax* epidemics broke out in the capital city [16, 19]. Since 2015, passive surveillance for malaria cases has been organized and followed over time at two sentinel sites. The objectives of the present study were to assess the trend of malaria prevalence in febrile patients consulting spontaneously at two health facilities in Nouakchott, based on reliable diagnostic tools, and to evaluate the current malaria situation. The findings of this study will be useful for the understanding of transmission dynamics of malaria, its evolution in time, and development of effective control strategies.

## Methods

### Study sites

**Fig. 1:**
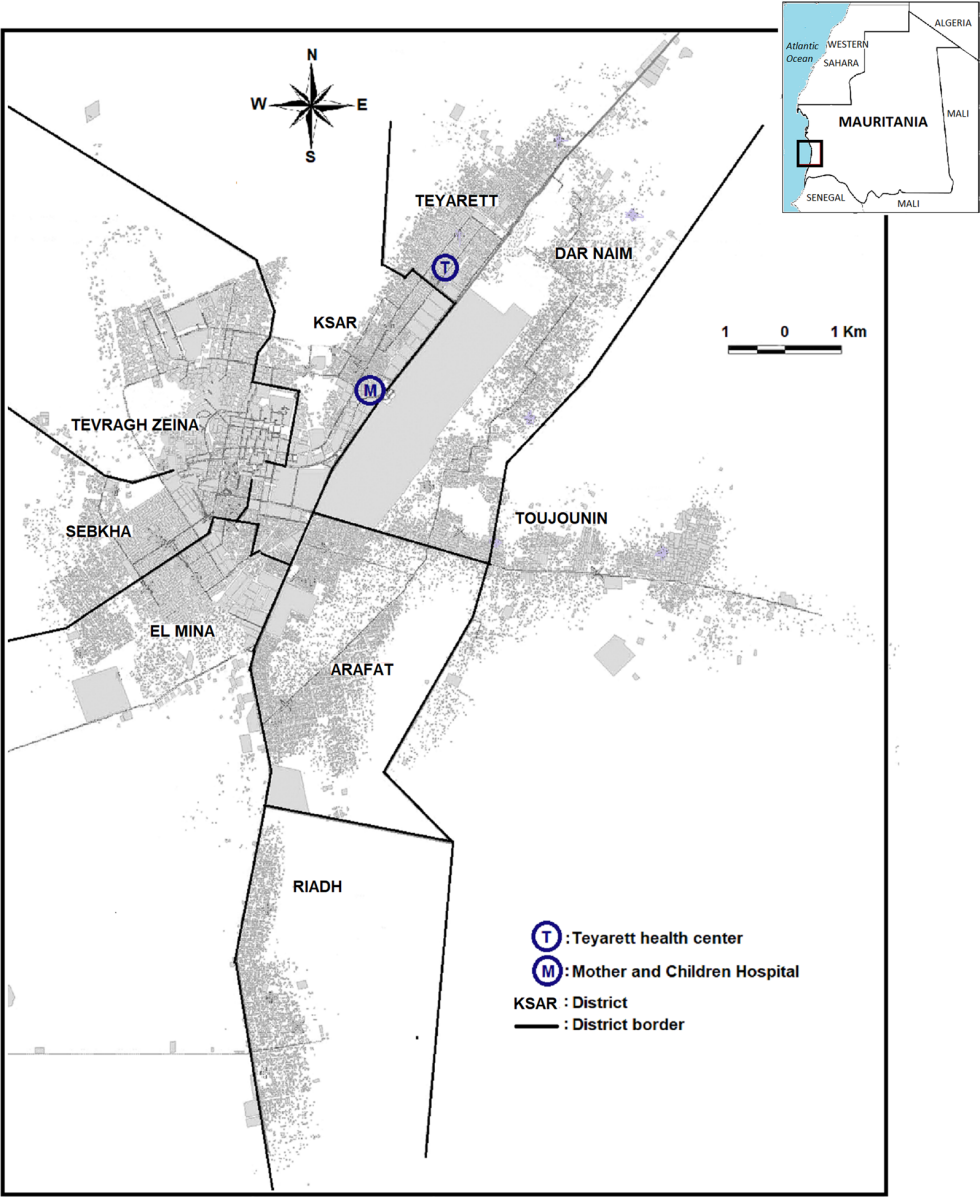
Map of Nouakchott, Mauritania, showing the location of the study sites: Teyarett health centre (T) in Teyarett district and Mother and Children Hospital Centre (M) in Ksar district. The names and limits of administrative districts are shown. The city centre is in Tevragh Zeina district, bordering Sebkha, Ksar, and El Mina districts. A large band of rectangular smooth gray area in Dar naim district bordering (M) and (T) represents the international airport (closed since 2016 due to the opening of the new international airport situated outside the city). The gray spots represent houses and buildings. The white zones are unoccupied desert area. The Atlantic Ocean lies to the west of the city

The study was carried out in Nouakchott (18°05′08″ North latitude and 15°58′42″ West longitude), situated along the Atlantic coast, characterized by a low elevation level ranging from − 1 m to 10 m above sea level (Fig. 1). A natural salty belt with a width of 1 to 2 km separates the city proper from the Atlantic coast. Since the beginning of the 2010s, a potable water supply system has been developed in the Saharan zone of the country through *Aftout Essahli* project, with the aim to transport and deliver treated surface water through water pipes from the Senegal River to city residents [20].

The climate in Nouakchott is characterized by a long dry season (October-June) and a short rainy season (July–September), with a mean annual rainfall of 89.5 mm for the period 1970–2013 [21]. During the study period (2015–2020), the mean annual rainfall was 79 mm (116 mm in 2015, 81 mm in 2016, 48 mm in 2017, 102 mm in 2018, 64 mm in 2019, and 62 mm in 2020). For comparison, the annual rainfall in the years when flooding occurred in Nouakchott was 195 mm in 2009, 174 mm in 2010, and 130 mm in 2013. The annual relative humidity ranged from 33 to 79%, and the average annual temperature ranged between a minimum of 20.5 °C and a maximum of 33 °C during the study period.

Nouakchott is densely populated with approximately 1 million inhabitants distributed across a surface area of 1000 km^2^ corresponding to a population density of 1000 inhabitants per km^2^. They belong to various ethno-linguistic groups, including White and Black Moorish group of Arab, Berber, and African ancestry speaking a common Arabic-derived dialect called “*Hassaniya*”, and the Pular, Wolof, and Soninké black African groups who speak *Pular*, *Wolof*, and *Soninké* languages, respectively.

### Study design and study population

This is a six-year, continuous, prospective monitoring study carried out during the period from January 1st, 2015 to December 31, 2020, in two health structures in Nouakchott: Teyarett health centre and the paediatric department of the Mother and Children Hospital Centre (“Centre hospitalier mère et enfant” CHME), located in the neighbouring districts of Teyarett and Ksar, respectively (see Fig. 1). Teyarett health centre mainly receives patients of all age groups from two districts in Nouakchott known to be highly endemic for malaria, Teyarett and Dar naim. The paediatric department of the CHME receives children and women from all districts of Nouakchott. These two health structures have been the sentinel sites of several clinical and epidemiological studies since 2010 [8, 16, 19, 22].

The study target population was outpatients in whom malaria infection was suspected, with either a measured axillary temperature > 37.5 °C at the time of consultation or history of fever within the previous 48 h with no other obvious cause, with no upper or lower age limit. The purpose of the study was explained in the local dialects to each adult patient and, for children under 18 years old, their parents or legal guardian, and written informed consent or oral assent was obtained before inclusion in the study. Since symptomatic patients were included in the study before laboratory confirmation of malaria diagnosis, the study population consisted of both malaria-positive and malaria-negative patients. An exclusion criterion was patients requiring emergency care, including severe and complicated malaria, who were not seen at the outpatient department of the two study sites but were seen and admitted in the emergency department.

After obtaining informed consent, finger-prick capillary blood samples were obtained to prepare thick and thin blood smears and perform rapid diagnostic test (RDT) for malaria. About 100 μL of capillary blood were spotted on Whatman 3MM filter paper and air dried (GE Healthcare Europe GmbH, Freiburg im Breisgau, Germany) for diagnosis by polymerase chain reaction (PCR). During medical consultation, each adult patient or one of the accompanying parents (or legal guardian) of the sick child under 18 years old was interviewed using a standard, pretested questionnaire covering socio-demographic data, including a detailed recent travel history outside Nouakchott and bed net ownership and use.

### Malaria diagnosis

Malaria parasite was detected using RDT, microscopic examination of blood smears, and PCR. RDT for malaria was performed using Standard Diagnostics Bioline malaria antigen Pf/Pan test (Standard Diagnostics/Abbott, Park, IL, USA) according to the manufacturer’s instructions. This RDT detects *P. falciparum*-specific histidine-rich protein 2 (PfHRP-2), referred to as the “Pf band,” and *Plasmodium* genus-specific lactate dehydrogenase (pLDH), referred to as the “Pan band.” Patients with RDT-positive result were treated, free of charge, with artemisinin-based combination therapy (ACT), according to the national policy for malaria case management recommended by the Mauritanian National Malaria Control Programme (NMCP).

Microscopic identification and quantification of asexual malaria parasites were performed using 5% Giemsa-stained blood smears according to the standard technical procedures recommended by the World Health Organization (WHO) [23, 24]. Asexual parasites were counted against at least 200 white blood cells (WBC) in thick smears, and parasite density was expressed as the number of asexual parasites/µL of blood, assuming 8000 WBC/µL of blood. Laboratory diagnosis based on RDT and microscopy was performed on-site to guide clinicians in selecting the most appropriate treatment.

The template DNA for parasite identification by PCR was extracted from dried filter paper using the Chelex method [25]. Molecular identification of *Plasmodium* species was determined retrospectively in the research laboratory of the University of Nouakchott by nested PCR protocol developed by Snounou et al. [26] to confirm the results of RDT and microscopy. The primer sequences used in the first (genus-specific) and second (species-specific) rounds of PCR amplification of the 18S ribosomal RNA gene and PCR conditions were published earlier [13]. DNA extracted from blood samples of confirmed cases of *P. falciparum*, *Plasmodium malariae*, *Plasmodium ovale* or *P. vivax* infection served as positive controls. The results of RDT, microscopy, and PCR were blinded with respect to each other. In the present study, laboratory diagnosis was limited to the detection of malaria parasites. Patients with a negative malaria test (microscopy and RDT) were referred to the attending physician for further diagnostic procedures. The clinical records of these malaria-negative patients were not available for further analysis of other infectious agents due to restrictions imposed by the consent form and ethical clearance limited to the study of malaria. Likewise, analysis of blood samples from patients with a positive malaria test (microscopy and/or RDT) was limited to malaria due to ethical constraints.

### Environmental data collection

Data on monthly rainfall were collected by the Mauritanian meteorological service and made available to the general public through the government meteorological office. Data concerning the evolution in the number of users connected to the water distribution network managed by National Water Distribution Company (“Société Nationale de distribution d’Eau” SNDE) and the public standpipes in Nouakchott before and after the launching of the *Aftout Essahli* project in 2010 were obtained from SNDE.

### Statistical analysis

Data were entered into an Excel spreadsheet (Microsoft Office Excel 2007, Microsoft Corporation, Redmond, WA). Descriptive statistics (frequencies, means, standard deviation, and percentages) of malaria prevalence (defined in this study as malaria positivity among febrile patients), rainfall pattern, and travel history was analysed using Microsoft Excel (Microsoft Corporation; Redmond, WA). The prevalence of malaria burden by sex, ethnic groups, and age groups, as well as the frequency of reported use of bed net, was compared by Fisher’s exact test using GraphPad InStat^®^ software, version 3 for Macintosh (GraphPad software Inc., San Diego, CA). The proportions of PCR-positive blood samples and the proportions of *P. vivax*-positive PCR over the successive years were compared by the chi-square test for trends using GraphPad InStat^®^. A simple linear regression analysis was performed after evaluating linearity (run test procedure) to assess the relationship between rainfall pattern and malaria prevalence using Microsoft Excel, GraphPad InStat, and GraphPad Prism 3, and Pearson’s correlation coefficient (r) was calculated. The level of significance was set at two-sided *P* < 0.05.

### Ethical considerations

The study protocol was reviewed and approved by the Institutional Ethics Committee of the Université de Nouakchott (formerly called Université de Nouakchott Al-Aasriya), Nouakchott, Mauritania (Approval No. 112/12-09-2014/USTM, 003/2020/CE/UNA) and the Institutional Ethics Review Board of the Institut de Recherche pour le Développement (IRD), Marseille, France (Comité consultatif de déontologie et d’éthique approval 15/12/2012). Informed written/oral consent from adult patients or the parents or legal guardians of children was obtained before blood collection.

## Results

### Characteristics of enrolled patients

Between January 2015 and December 2020, a total of 1,760 adult and paediatric febrile patients with a clinical suspicion of malaria were screened on-spot for malaria using RDT and microscopic examination of blood smears and later confirmed by PCR. The numbers of screened patients per year varied between 103 (5.8%) in 2017 and 459 (26.1%) in 2015 (Table 1).

**Table 1:** Characteristics of patients with a clinical suspicion of malaria included in the study

Characteristics^a^	2015	2016	2017	2018	2019	2020	2015–2020
N (%)	459 (26.1)	325 (18.5)	103 (5.8)	192 (11.0)	402 (22.8)	279 (15.8)	1760 (100)
Sex							
Male	258 (56.2)	173 (53.2)	57 (55.3)	108 (56.3)	171 (42.5)	99 (35.5)	866 (49.2)
Female	201 (43.8)	152 (46.8)	46 (44.7)	84 (43.7)	231 (57.5)	180 (64.5)	94 (50.8)
Age group (yrs)							
< 5	68 (14.8)	141 (43.4)	54 (52.4)	64 (33.3)	61 (15.2)	106 (38.0)	494 (28.0)
5–9	67 (14.6)	47 (14.4)	11 (10.6)	14 (7.3)	21 (5.2)	16 (5.7)	176 (10.0)
10–14	53 (11.5)	37 (11.4)	14 (13.6)	35 (18.2)	66 (16.4)	46 (16.5)	251 (14.3)
15–19	45 (9.8)	10 (3.1)	2 (2.0)	23 (12.0)	53 (13.2)	19 (6.8)	152 (8.6)
≥ 20	226 (49.2)	90 (27.7)	22 (21.3)	56 (29.1)	201 (50.0)	92 (32.9)	687 (39.0)
Ethnic group^b^							
White Moors	374 (81.5)	236 (72.6)	72 (69.9)	128 (66.6)	264 (65.7)	189 (67.7)	1263 (71.8)
Black Moors	62 (13.5)	69 (21.2)	24 (23.3)	55 (28.6)	105 (26.1)	77 (27.6)	392 (22.3)
Black Africans	23 (5.0)	20 (6.1)	7 (6.8)	9 (4.7)	33 (8.2)	13 (4.6)	105 (5.9)

^a^Data are expressed as the number (N) and % in parentheses. The last column (2015–2020) presents the total number (%) of febrile patients with the corresponding characteristics

^b^The group referred to as “black Africans” denotes those who belong to Pular, Wolof, or Soninké ethnic groups, each of whom is characterized by a distinct dialect (Pular, Wolof, and Soninké dialects, respectively)

The majority of the participants (687/1760; 39%) were adults aged ≥ 20 years, followed by children aged < 5 years old (494/1760; 28%). The age of the study population ranged from 1 month to 87 years old. The proportion of females (50.8%; 894/1760) was closely similar to that of males (49.2%; 866/1760) during the six-year study period despite the fact that one of the sentinel sites (CHME) was specialized for mothers. The sex-ratio was 0.97. The majority of screened patients (71.8%; 1263/1760) belonged to the white Moorish ethnic group. Patients of black Moorish or black African ancestry (Pular, Soninke and Wolof) represented 22.3% (392/1263) and 5.9% of the study population, respectively.

### Malaria prevalence, *Plasmodium* species, and travel history

The global prevalence of malaria (i.e. malaria positivity among febrile patients) based on the results of RDT, microscopy, and PCR was 15.5% (274/1760), 14.5% (256/1760), and 16.5% (291/1760), respectively, during the study period 2015–2020 (Table 2). As expected, PCR detected the highest number of malaria-positive samples. Among 291 PCR-positive cases, 216 (74.2%) were due to *P. vivax*, 47 (16.1%) to *P. falciparum*, and 28 (9.6%) to *P. falciparum-P. vivax* mixed infections. *Plasmodium malariae* and *P. ovale* were not observed among PCR-positive samples. There was a statistically significant decline in malaria prevalence based on PCR from 2015 to 2020 (*χ*^2^ for trend, 88.3; 1 degree of freedom; *P* < 0.0001). However, analysis of the proportions of *P. vivax*-positive (monoinfection *P. vivax* or mixed *P. falciparum*-*P. vivax* infection) PCR to PCR-positive *P. falciparum* monoinfection did not show any significant linear trend during the study period (*χ*^2^ for trend, 0.0000751; 1 degree of freedom; *P* = 0.993), suggesting the predominance of *P. vivax* over *P. falciparum* during the entire study period. The geometric mean parasite density per year varied between 1100 asexual parasites/μl of blood in 2018 and 3060 asexual parasites/μl of blood in 2017, with an overall geometric mean of 1480 asexual parasites/μl of blood during the entire study period (2015–2020).

**Table 2:** Malaria prevalence, *Plasmodium* species, and parasite density in Nouakchott (2015–2020)

Characteristics^a^	2015	2016	2017	2018	2019	2020	2015–2020
N (%)	459 (26.1)	325 (18.5)	103 (5.8)	192 (11.0)	402 (22.8)	279 (15.8)	1760 (100)
Malaria-positive							
RDT	166 (36.1)	63 (19.3)	5 (4.8)	23 (11.9)	17 (4.2)	0	274 (15.5)
Microscopy	131 (28.5)	52 (16.0)	5 (4.8)	33 (17.1)	34 (8.4)	1 (0.3)	256 (14.5)
PCR	134 (29.2)	64 (19.7)	5 (4.8)	29 (15.1)	53 (13.2)	6 (2.1)	291 (16.5)
*Plasmodium* spp.							
Pv	107 (79.8)	30 (46.9)	5 (100)	21 (72.4)	49 (92.5)	4 (66.6)	216 (74.2)
Pf	18 (13.4)	15 (23.4)	0	8 (27.6)	4 (7.5)	2 (33.3)	47 (16.1)
Pf–Pv	9 (6.7)	19 (29.7)	0	0	0	0	28 (9.6)
Parasite density	1250	2540	3060	1100	1730	935	1480

^a^Data are expressed as the number (N) and % in parentheses (the denominator is the total number of screened patients per year shown in the first row) for malaria prevalence determined by different diagnostic tools and *Plasmodium* species determined by PCR (Pv, *P. vivax* monoinfection; Pf, *P. falciparum* monoinfection; Pf-Pv, *P. falciparum*-*P. vivax* mixed infection). The last column (2015–2020) presents the total number (%) of malaria-positive patients with each diagnostic tool and distribution of *Plasmodium* species during the entire study period (2015–2020). PCR did not detect any *P. ovale* or *P. malariae*. Parasite density denotes the geometric mean parasite density, expressed as the number of asexual parasites per µL of blood. The parasite density in 2020 (935 asexual parasites/µL) is based on a single smear-positive sample

Of 1760 included patients, information on travel history was available in 1658 (102 missing data, 10 from patients with PCR-positive *P. falciparum*-*P. vivax* mixed infections and 92 from PCR-negative patients). As the endemicity of *P. vivax* malaria in Nouakchott has already been established since two decades [7], in this study, the travel history of *P. falciparum*-positive patients, either as a monoinfection or mixed *P. falciparum*-*P. vivax* infection, was analysed (Table 3). Of 47 patients diagnosed with *P. falciparum* monoinfection during the study period (2015–2020), 29 (61.7%) reported a recent journey (i.e., less than 1 month before consultation) to the southern regions of the country where *P. falciparum* malaria is known to be endemic. Two of 29 patients returned from a trip to Côte d’Ivoire and Mali, where *P. falciparum* malaria is highly endemic, and transmission is generally stable throughout the year. However, 18 of 47 (38.3%) patients, including four children under 3 years old, had not travelled outside Nouakchott, suggesting a probable local transmission of *P. falciparum* in Nouakchott itself. Of 28 patients with mixed *P. falciparum*-*P. vivax* infections, information on travel history was available in 18 of them, of whom 16 (88.9%) had a travel history to various regions in the country, including 2 patients who had recently visited Nouadhibou in the northern Saharan zone where there are no reliable data on malaria epidemiology. The results suggested a statistically significant association (relative risk, 9.346; 95% confidence interval, 7.370–11.852; *P* < 0.0001) between a recent travel history (i.e. < 1 month before consultation) and *P. falciparum* monoinfection or *P. falciparum-P. vivax* mixed infection, compared to those with either *P. vivax* monoinfection or PCR-negative result.

**Table 3:** Travel history of patients with PCR-confirmed malaria

*Plasmodium* spp.	Travel < 1 mo	Travel > 1 mo	No travel	Total
Pf	29^a^	0	18	47
Pf–Pv	16^b^	1	1	18^c^
Pv	10	48	158	216
PCR-negative	108	121	1148	1377^c^
Total	163	170	1325	1658

Patients were classified as those with PCR-confirmed *P. falciparum* monoinfection (Pf), those with PCR-confirmed *P. falciparum*–*P. vivax* mixed infection (Pf–Pv), *P. vivax* monoinfection (Pv), and PCR-negative. Data are expressed as the number of patients with or without travel history outside Nouakchott. Travel history was classified as “no travel” for patients who had never travelled outside Nouakchott, “travel < 1 mo” for patients who had travelled outside Nouakchott any time within one month before consultation, and “travel > 1 mo” for patients who had travelled outside Nouakchott, including foreign countries, more than one month before consultation

^a^Regions where the patients travelled to include Hodh Echarghi (n = 14), Hodh Elgharbi (n = 7), Brakna (n = 2), Assaba (n = 2), Trarza (n = 1), and Nouadhibou (n = 1). One patient travelled to Côte d’Ivoire; another travelled to Mali

^b^Regions visited by these patients before consultation include: Hodh Echarghi (n = 5), Hodh Elgharbi (n = 2), Trarza (n = 4), Nouadhibou (n = 2), Guidimagha (n = 1), Gorgol (n = 1), and Tagant (n = 1)

^c^Information on travel history was missing in 10 of 28 patients with *P. falciparum*-*P. vivax* mixed infections and in 92 of 1469 PCR-negative individuals

### Prevalence of *P. vivax* in relation to age, sex, and ethnic groups

The number of PCR-confirmed *P. vivax* monoinfections by age, sex, and ethnic group during the study period is summarized in Table 4. There were a total of 216 PCR-positive *P. vivax* monoinfections. Adults ≥ 20 years were most affected (110/216; 51.0%), followed by the age group of 10–14 years old (33/216; 15.3%) and under five children (32/216; 14.8%). Analysis of data by two categories, adults (> 20 years old) versus children and adolescents (< 20 years old), suggested a higher proportion of adults infected with PCR-confirmed *P. vivax* (110/687; 16.0%) than in children and adolescents (106/1073; 9.9%) (*P* = 0.0002). Of 216 PCR-confirmed *P. vivax* malaria cases, 128 (59.3%) and 88 (40.7%) were in males and females, respectively. The difference in the proportions of PCR-positive *P. vivax* between males and females was statistically significant (*P* = 0.0018), indicating that a higher proportion of males were infected with *P. vivax*. White Moors were relatively more affected in number by *P. vivax* monoinfection (166/1760 total sample population; 9.4%) than black Moors and black Africans pooled together (50/1760; 2.8%) due to their overrepresentation in this patient population, but this difference in proportions was not statistically significant (*P* = 0.0765). Separate analysis showed that the difference in the proportions of PCR-positive *P. vivax* was not statistically significant between white Moors (166/1263, 13.1%) and black Moors (44/392; 11.2%) (*P* = 0.340), but the difference was statistically significant between white Moors (9.4%) and black Africans (6/99; 6.1%) (*P* = 0.0305). The present study showed that male adults belonging to white or black Moor ethnic groups were the most affected by *P. vivax* monoinfection.

**Table 4:** *P. vivax* monoinfections by age, sex, and ethnic groups in Nouakchott, 2015–2020

Characteristics	PCR-positive *P. vivax* infections
Age group (years)	
< 5	32 (14.8)
5–9	18 (8.3)
10–14	33 (15.3)
15–19	23 (10.6)
≥ 20	110 (51.0)
Total	216
Sex	
Male	128 (59.3)
Female	88 (40.7)
Ethnic group	
White Moors	166 (76.8)
Black Moors	44 (20.4)
Black Africans	6 (2.8)

Data are expressed as the number of PCR-confirmed *P. vivax*-infected patients and % in parentheses. There were a total of 216 PCR-confirmed *P. vivax* monoinfections

For statistical analysis, the age groups < 5, 5–9, 10–14, and 15–19 years old were pooled and considered as “children and adolescents” for comparison to “adults” (> 20 years old). The difference in the proportions of *P. vivax* monoinfections in children and adolescents (106/1073, 9.9%) and in adults (110/687, 16.0%) was statistically significant (*P* = 0.0002). The difference in the proportions of males and females with *P. vivax* was statistically significant (*P* = 0.0018)

The ethnic groups, Black Moors and black Africans, were pooled together into a single group and compared to white Moors. The difference was not statistically significant (*P* = 0.0765). The ethnic groups were also compared separately, 2 by 2. The difference in the proportion of white Moors infected with *P. vivax* monoinfection (9.4%) and that of black Africans with *P. vivax* monoinfection (6.1%) was statistically significant (*P* = 0.0305). There was no statistically significant difference between white and black Moors (*P* = 0.340)

### Monthly and annual malaria prevalence

**Fig. 2:**
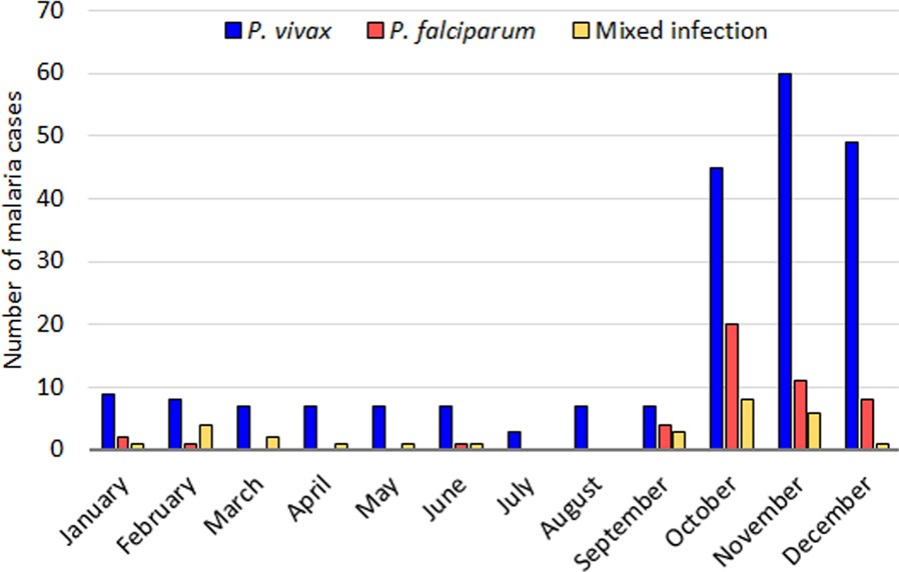
Monthly distribution of PCR-positive malaria-infected patients in Nouakchott. Data from 2015–2020 were pooled by month

The monthly prevalence of malaria infections over a six-year period suggested that *P. vivax* monoinfections occurred throughout the year, but they were more frequent towards the end of the rainy season (i.e., September) and for a few months following the end of the rainy season (October, November and December), with the peak occurring in November (Fig. 2). By contrast, *P. falciparum* cases were detected mostly at the end of the rainy season (September) and the peak transmission occurred in October.

**Fig. 3:**
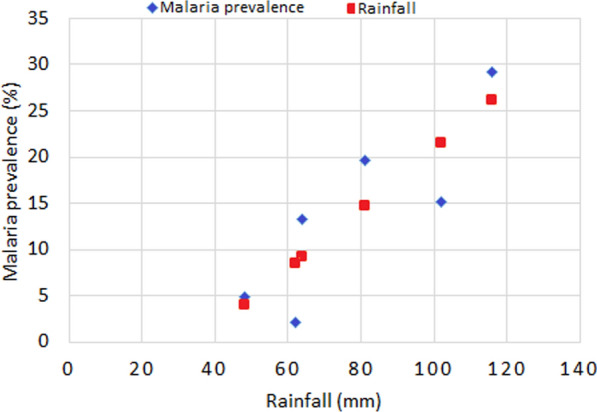
Trend of malaria prevalence and rainfall pattern in Nouakchott from 2015 to 2020. The annual rainfall and the annual prevalence of PCR-confirmed malaria showed a linear relationship (probability of departure from linearity, *P* = 0.900) and a statistically significant correlation (Pearson correlation coefficient r = 0.853; goodness of fit, r^2^ = 0.727; two-tailed *P* = 0.031; $y=2.235x+47.511$)

The analysis of yearly data showed that the proportions of malaria positivity consistently declined from 29.2% (134/291) in 2015 to 2.1% (6/291) in 2020 (Table 2). During the same period, the amount of annual rainfall also decreased from 116 mm in 2015 to 62 mm in 2020. Regression analysis showed that a linear model is appropriate for the data (probability of departure from linearity, *P* = 0.900), and based on the best-fit values, a strong positive correlation between the amount of rainfall and malaria prevalence was observed (Pearson correlation coefficient, r = 0.853; r^2^ = 0.727; *P* = 0.031), suggesting an association between decreasing amount of rainfall and decreasing malaria prevalence from 2015 to 2020 in Nouakchott (Fig. 3).

### Bed net ownership and use

Of 1760 patients, information on bed net use was available from 582 (33%) of them. Almost three times as many febrile patients reported that they do not own or use bed nets (426/582; 73.2%) as those who reported to own and use bed nets that were either treated with insecticide or not (156/582; 26.8%). The proportions of malaria-positive patients who reported to be bed net users (25.0%; 39/156) and those who were malaria-positive and non-users of bed nets (20.6%; 88/426) did not reach a significant difference (*P* = 0.2597). Because of the large number of missing data, however, the information provided here may not be very reliable.

## Discussion

The present work is a follow-up longitudinal study spanning over six years on the evolution of malaria prevalence among febrile patients consulting spontaneously at two sentinel sites in Nouakchott. The majority of patients with PCR-confirmed malaria were due to *P. vivax*, which occurred more frequently in adult males ≥ 20 years old, in agreement with the results reported in earlier studies conducted in Nouakchott [8, 12, 13, 22]. The difference in malaria prevalence between age groups and sex is probably associated with behavioural and occupational differences, but studies on social and behavioural aspects related to malaria risk and knowledge, attitude, and perception survey have not been performed in Mauritania to explain this difference. Although the current proportions of different ethnic groups are not known in the country, white Moors (71.8% vs 22.3% black Moors and 5.9% black Africans) were overrepresented in the patient population of the present study. White Moors were affected more frequently (76.8%) by *P. vivax* malaria than the other ethnic groups present in the country. This finding confirms that of earlier studies conducted not only in Nouakchott, but also in other sentinel sites in the country [9, 15, 22]. The results are consistent with the hypothesis that Duffy antigen is the major receptor necessary for the entry of *P. vivax* merozoites into reticulocytes and with the fact that > 50% of white Moors are Duffy-positive and 98% of black Africans are Duffy-negative [27, 28]. Furthermore, the data presented in the present study also suggested that *P. vivax* can also infect black Moors and black Africans, the majority of whom are known to be Duffy-negative [27, 29]. Further studies would be necessary to update epidemiological data on Duffy antigen in different ethnic groups in Mauritania and evaluate to what extent the presence of Duffy antigen is required for *P. vivax* malaria to occur.

The update presented in the present study fills an important gap in the epidemiological data on malaria in Mauritania and highlights the current malaria situation in the capital city of Mauritania. For centuries, Nouakchott had been a small fishing village with no particular history attached to it. Until the accession of Mauritania to independence in 1960, there were at most 500 permanent residents in Nouakchott (more precisely, in “Ksar,” a historical district of Nouakchott) [30]. Nouakchott was chosen as the capital of a new nation in 1960. Since then, the process of urbanization in Nouakchott has been intense and unbridled, to the point of becoming by far the largest city, not only in Mauritania, but also in the Sahara Desert today [31, 32]. The rapid pace of urbanization in Nouakchott was largely fueled by a massive rural exodus following the major long-lasting drought in the Sahel region in southern Mauritania during the 1970s and 1980s, resulting in an overcrowded city and leading to multiple challenges in urban organization. One of the most pressing problems that confronted the newly independent nation since 1960 concerned both scarcity and quality of safe potable water supply for the rapidly increasing urban populations. To palliate this problem, several public potable water fountains have been established to supply clean water that was pumped from the Idini aquifer’s well field, 60 km east of Nouakchott to newly settled populations residing in the slums surrounding the city centre. However, inadequate management of these water points have created stagnant water around them, resulting in the creation of breeding sites for mosquitoes, particularly *Anopheles gambiae *sensu lato (*s.l*.), but also *Aedes aegypti* [18, 33]. To bring about a more permanent solution to the problem of chronic water shortage in the capital city with an ever-growing population, in 2010, the Mauritanian government launched the *Aftout Essahli* project. While the project is at present in an advanced stage of implementation in Nouakchott and undoubtedly represents a major achievement for many Nouakchott residents, on the negative side of the ledger, the higher pressure and the age of the network have also increased the number of leaks and creation of additional mosquito larval habitats [18, 34].

During the last two decades, several studies had been conducted to assess the epidemiology of malaria in Nouakchott, mostly during the period between 2007 and 2013, which coincides with the commencement of the *Aftout Essahli* project. Some reported a high prevalence of malaria among febrile patients in health facilities in Nouakchott [8, 13, 22]. However, there has been no follow-up study since 2013 to produce an accurate and comprehensive estimate of malaria prevalence in the city, particularly after the completion of the *Aftout Essahli* project. The results of the present study address and fill this data gap, at least in part.

The longitudinal data from 2015 to 2020 suggest several key epidemiological features: (i) after the occurrence of a high prevalence of *P. vivax* infections to “epidemic proportions” in 2012–2013 among febrile patients, there is a clear trend towards a declining prevalence due to malaria (both *P. vivax* and *P. falciparum*) from 2015 onwards, even though the decline is not uniform year to year; (ii) *P. vivax* still remains predominant in Nouakchott; (iii) both *P. falciparum* and *P. vivax* malaria occur during a relatively short, well-defined period, i.e. at the end of, and just after the rainy season (September–December), but only *P. vivax* continues to be present throughout the rest of the year during the dry season, with the exception of very few cases of *P. falciparum* infection; and (iv) surprisingly, despite the absence of an entomological proof at present, the data presented in this study seem to suggest a local transmission of *P. falciparum*.

The two sentinel sites where many of the previous works on malaria had been conducted draw patients from the districts (Teyarett and Dar naim districts) where *Anopheles* mosquitoes abound and *P. vivax* epidemics were notified and demonstrated in 2012–2013 [7, 35, 36]. In an earlier study conducted in Nouakchott in 2009–2010, it was reported that 34.9% (105 of 301) of samples from febrile children included in three health centres, including Teyarett health centre, were PCR-positive (97.1% *P. vivax* and 2.9% *P. falciparum*) [22]. During the 2012–2013 malaria epidemics, almost 5,000 cases, representing 54% of clinically suspected malaria cases, were diagnosed as *P. vivax* malaria by RDT in Teyarett health centre alone, mostly during the months of September or October to December [16]. In that study, only *P. vivax* was detected, despite the use of government-approved PfHRP2/pLDH-based RDT and confirmation by PCR.

In another study conducted during the same period (2015–2016) as the present study in Atar, a city located in the Saharan zone to the north of Nouakchott, the performance of the RDT was assessed in comparison to nested PCR [9]. Malaria epidemiology in Atar is similar to that of Nouakchott, with a clear predominance of *P. vivax* over *P. falciparum*. The sensitivity and the specificity of the RDT to detect *P. vivax* were 63% and 99%, respectively. Despite the relatively low sensitivity to detect *P. vivax* using RDT, most probably due to generally low parasitaemia encountered in Mauritanian patients, data interpretation in the present work was based on the results of PCR to offset this technical limitation.

Based on the assumption that similar types of patients were screened and included in the present study from the same sentinel sites, it can be observed that the number of symptomatic patients with a clinical suspicion of malaria declined considerably in 2015–2020 (range, 103 to 459 patients per year; see Table 2 for details) despite a continuous, year-long monitoring for 6 years. More importantly, the mean proportion of PCR-confirmed malaria declined from 34.9% in 2009–2010 to 16.5% in 2015–2020. Several plausible explanations can be given.

First, as shown in the analysis of malaria prevalence and the amount of annual rainfall, a climate change characterized by a relatively reduced rainfall during the period 2015–2020 is highly correlated with decreasing malaria prevalence among febrile patients. By contrast, the unusually high amount of precipitation recorded in Nouakchott in 2009, 2010, and 2013 (130–195 mm, compared to the average annual precipitation of 79 mm in 2015–2020) probably contributed to increase malaria transmission in the early 2010s. In addition, floods occurred in the city in 2010 and 2013 [37]. Because of the absence of a water evacuation system, the city remained flooded for several weeks, or even months in some districts, during those years. The 2012–2013 *P. vivax* epidemics in Nouakchott can at least in part be ascribed to the large-scale flooding of the city just before the malaria transmission season [16]. Unusual variations and decrease in the annual rainfall have been recorded in Nouakchott in 2015–2020. The climate change, as well as the rising water table resulting in salty water pools seeping through the soil in areas below sea level, had a direct influence on the stability and productivity of anopheline larval habitats in Nouakchott, favouring the creation of highly saline water bodies [37]. Although some *An. arabiensis* populations have adapted to saline habitats, overall, the increased salinity resulted in decreased anopheline mosquitoes.

Second, a new water distribution system was installed in the capital city during the 2010s. In the early 2010s, public standpipes and water distribution using water tanks drawn by donkeys (a common scene in Teyarett-Dar naim districts) have been directly implicated in the creation of artificial mosquito larval habitats in these districts [18]. Despite initial technical problems leading to frequent leakage few years after the launching of the *Aftout Essahli* project in 2010, the completion of the potable water supply project in Nouakchott resulted in a considerable reduction in the number of public standpipes in Teyarett, Dar naim, and Ksar districts from 72 in 2013 (9 in Ksar, 23 in Teyrett, and 40 in Dar naim) to only 29 (i.e. 59.1% decrease) in 2020 (1 in Ksar, 10 in Teyarett and 18 in Ksar). During the same period, there was an overall increase in the number of households connected to the SNDE water supply network in Nouakchott, from 53,423 in 2013 to 135,723 in 2020, corresponding to an increase of 60.6%. The expansion and improvement of water distribution network, possibly along with increasing environmental pollution, have been associated with changing distribution of mosquito species. An epidemiological study conducted at that time showed that about 39–44% of indoor resting mosquitoes captured by pyrethrum spray catch in the morning or captured by light traps at night in Teyarett district in 2011–2013 belonged to *Anopheles* spp. (the rest of the specimens were *Culex* spp. or *Aedes* spp.) [14]. By contrast, in an entomological survey conducted at several sites near Teyarett and Dar naim districts in Nouakchott in 2016–2017, only 2.5% (41/1600) of indoor resting mosquitoes were *An. arabiensis* (the others included *Aedes aegypti* [26%], *Anopheles multicolor* [0.7%], and *Culex* spp. [70.7%]) [37]. The available entomological data tend to support the observation that the proportion of *An. arabiensis* among captured mosquito specimens in Nouakchott seems to have decreased during the 2010s.

Third, the Mauritanian Ministry of Agriculture and Ministry of Health had carried out a mass campaign of outdoor residual spraying of insecticides in different districts of Nouakchott in 2015 and 2016 in response to the mounting levels of complaints from residents due to nuisance caused by mosquitoes and houseflies [38]. Although numerous anecdotal accounts of the residents have suggested that spraying has resulted in a drastic diminution of mosquitoes and houseflies, unfortunately, rigorous scientific studies designed to measure the impact of spraying were not performed.

Fourth, because well-trained microscopists are rare in the country, the use of RDT has been resorted to massively since its introduction to the country in the early 2010s. The supply of RDT and ACT is subsidized through the Global Fund, and RDT-based diagnosis, as well as ACT for RDT-positive patients, is supposed to be provided free of charge to patients consulting public health centres [39]. The real impact of Global Fund programme for Mauritania has not been evaluated, precluding the possibility of drawing any conclusion with regards to the improvement of diagnosis and treatment as one of the possible causes of declining malaria prevalence in Nouakchott.

Fifth, another factor to take into consideration is the government initiative for a mass distribution of insecticide-impregnated bed nets and increased awareness of the residents through health education, possibly leading to an increased use of bed nets to protect pregnant women and young children and thereby reduce malaria prevalence. This initiative is also subsidized by the Global Fund, but its impact has not been assessed. In reality, however, bed net distribution in Nouakchott has been limited to pregnant women during a limited period of campaign period. This observation is indirectly supported by the results of the present study in which many of the participants (426/582, 73.2%), who did not include pregnant women, reported that they do not use or even own a bed net. Despite the emergence of malaria in Nouakchott and subsequent increase in prevalence during the 2000s and early 2010s [40], the Mauritanian NMCP has decided to distribute bed nets in priority areas situated in southern Mauritania, outside Nouakchott. However, even in the Sahelian zone with high prevalence of *P. falciparum*, close to one-third of febrile patients reported that they did not own or use bed nets in their household, and the reported use of bed nets did not reduce the probability of being infected by malaria [10]. The limited data available indicate that the coverage of bed net distribution has not reached an adequate level to have any impact on malaria prevalence in the country.

Sixth, another possible cause of the declining proportion of febrile illness attributable to laboratory-confirmed malaria is the emergence of other febrile illnesses that may mimic the clinical presentation of uncomplicated malaria in Nouakchott. For example, dengue fever was unknown in Nouakchott before 2014. In 2014 the presence of mosquito vector of dengue virus, *Aedes aegypti*, was identified for the first time in Nouakchott [33]. In the same year, the first known dengue epidemic broke out in Nouakchott [41]. According to anecdotal evidence, some Nouakchott adult residents have had febrile illness with a negative result for RDT for malaria but positive RDT for dengue. Furthermore, since 2014, dengue epidemics have been reported almost annually from different parts of the country. Another example is coronavirus disease 2019 (COVID-19) pandemic due to severe acute respiratory syndrome coronavirus 2 (SARS-CoV-2). The country has been affected by COVID-19 epidemic from March 2020 [42]. According to the data presented in Table 2, blood samples collected in 2020 had the lowest prevalence (2.1%) of PCR-confirmed malaria during the study period (2015–2020). Unfortunately, malaria RDT-negative febrile patients were not followed and medical information of the patients included in the present study on possible SARS-CoV-2 viral infection is not available since the latter was not covered by the study protocol and lied outside the scope of the ethical clearance and informed consent.

The reduction of malaria prevalence in Nouakchott in 2015–2020 can be ascribed mostly to the complex interaction of ecological, environmental, and meteorological modifications, with possibly an unknown impact due to the outdoor residual spraying in 2015–2016, more than due to specific malaria interventions based on prevention, diagnosis, and treatment. As observed in earlier studies conducted in Nouakchott [13, 14, 22], *P. vivax* is still the predominant malaria species in the capital city with no obvious change in the tendency of *P. vivax*-to-*P. falciparum* ratio (roughly 3:1 or 75% *P. vivax*). In Nouakchott, until proven otherwise, malaria parasites are transmitted exclusively by *An. arabiensis*, which can transmit both *P. vivax* and *P. falciparum*, as demonstrated in other African countries where *P. vivax* and *P. falciparum* are sympatric [43–45]. It is therefore not easy to understand why *P. falciparum* has been responsible for only a minority of malaria infections in Nouakchott until present. Elsewhere in southern Sahelian zone of the country (e.g., Kobeni, Kiffa, Rosso), where *An. arabiensis* is also the major malaria vector, *P. falciparum* predominates [8, 10, 11, 14, 46, 47].

Earlier works performed in Nouakchott in 2009–2013 have reported either total absence or few cases of *P. falciparum* in the capital city [8, 13, 14, 16, 22]. Moreover, the peak transmission period for *P. falciparum* was consistently 1 month earlier (i.e. in October) than that of *P. vivax* (peak in November) during the six-year study period. This time offset corresponds to the rainy season in the Sahelian south which begins earlier (June or July) than in the Saharan zone where Nouakchott is situated (August or September) [10]. This observation is also in agreement with (i) the results of the present study which showed that the majority of *P. falciparum*-infected patients (69%, including *P. falciparum*-*P. vivax* mixed infections) reported travel to regions in southern Mauritania where *P. falciparum* (also *P. vivax* to a lesser extent) is endemic less than 1 month before consultation and (ii) also with the hypothesis that *P. falciparum* malaria is allochthonous in Nouakchott. This unproven hypothesis is plausible due to the regular travels of local populations to and from the capital city to localities in southern region, both for personal reasons related to family ties and business. The road transportation system that links the capital city to various cities and towns in *P. falciparum* endemic zone is not dense and extensive, but it is efficient for relatively rapid transportation. However, on the contrary, the data in the present study also suggest the occurrence of a local transmission of *P. falciparum* in Nouakchott. Close to one-third of patients with PCR-confirmed *P. falciparum*, alone or mixed with *P. vivax*, denied having travelled outside Nouakchott. Because *P. falciparum* is undoubtedly present in Nouakchott and few cases may be mixed *P. falciparum*-*P. vivax* infections, which may possibly be misdiagnosed by RDT or microscopy, an accurate diagnosis of malaria species and appropriate treatment are required even though, from the medical and ethical viewpoint, a pragmatic approach to case management may be to consider all laboratory-confirmed malaria cases as potentially fatal falciparum malaria. Based on the available evidence, it can be hypothesized that *P. falciparum* malaria diagnosed in Nouakchott has been cases of imported malaria from the Sahelian southern regions of the country and that this malaria species is still in the process of becoming endemic in the capital city. Further studies will be required to prove this hypothesis, in particular by detecting *P. falciparum* in the salivary glands of *An. arabiensis* captured in Nouakchott. In addition to entomological surveys, a long-term regular parasitological surveillance would be necessary to assess malaria burden due to *P. falciparum* in Nouakchott.

The surveillance system to gather epidemiological data, as described in this work, has several limitations. The evaluation of malaria burden was limited to symptomatic patients presenting spontaneously at the sentinel sites in Nouakchott. Because patient recruitment was performed in an out-patient setting, a possible occurrence of severe and complicated *P. vivax* malaria was not evaluated in the present study. In addition, in a passive surveillance the malaria situation in the general population in Nouakchott, in particular asymptomatic relapse or carriage of malaria parasites, cannot be evaluated. The patient’s clinical record in health centres and hospitals is not computerized, and the patients do not possess any written clinical record of past illnesses. A patient’s past history on malaria infections and information on possible malaria reinfections or relapse during the six-year surveillance period cannot be assessed. Moreover, data between March and July 2020 are incomplete due to the temporary suspension of patient recruitment during the national lockdown decreed by the government to limit the propagation of SARS-CoV-2 virus responsible for COVID-19 pandemic. However, since the period of lockdown lies outside the malaria transmission period, the missing data in 2020 would likely have a limited impact on the analysis of data in the present work. On the contrary, the strengths of the present study lie on a longitudinal surveillance in the same sentinel sites as in the past studies. The inclusion criteria of the patients were the same as those of the past studies. Laboratory diagnosis was confirmed by PCR. Despite the inherent weakness of a passive disease surveillance, the present study provides various insights into the dynamics of malaria transmission in Nouakchott in recent years.

## Conclusions

The longitudinal malaria surveillance in Nouakchott between 2015 and 2020 highlights a declining prevalence among febrile patients of all ages, the predominance of *P. vivax* over *P. falciparum*, in particular among adult male white or black Moors, and a marked seasonal transmission pattern of both *P. vivax* and *P. falciparum* during and shortly after the rainy season, followed by ‘residual’ *P. vivax* infections, but not *P. falciparum* infections, throughout the remainder of the year. These changes in malaria epidemiology in Nouakchott are most likely due to climate change (i.e., diminished rainfall) and better management of the newly established potable water supply network. For greater impact of malaria control leading to malaria elimination, a more rational deployment of preventive, diagnostic, and therapeutic means, accompanied by regular evaluation of health and malaria indicators and measurement of impact, is necessary. A shift in control strategy designed to eliminate *P. vivax*, including the introduction and use of 8-aminoquinolines (primaquine, tafenoquine) should be considered. Moreover, chloroquine efficacy, demonstrated to be very high in 2013 in Nouakchott [19], should also be re-evaluated for its possible re-introduction for the treatment of *P. vivax* monoinfections in the country.

## Data Availability

All data generated or analysed during this study are included in this published article.

## Abbreviations

ACT: Artemisinin-based combination therapy
CHME: Centre hospitalier mère-enfant (Mother Child Hospital Centre)
COVID-19: Coronavirus disease 2019
PfHRP2: *Plasmodium falciparum* Histidine-rich protein 2
IRD: Institut de Recherche pour le Développement
LLIN: Long-lasting insecticide-impregnated nets
NMCP: National Malaria Control Programme
PCR: Polymerase chain reaction
pLDH: Plasmodial lactate dehydrogenase
RDT: Rapid diagnostic test
SNDE: Société Nationale d’Eau (National Water Distribution Company)
WBC: White blood cells
WHO: World Health Organization
